# Light-Chain Amyloidosis: The Great Impostor

**DOI:** 10.3390/life14010042

**Published:** 2023-12-26

**Authors:** Georgia Stefani, Evangelia Kouvata, George Vassilopoulos

**Affiliations:** 1 Department of Hematology, Larisa University Hospital, 41110 Larisa, Greece; grg.stefani@gmail.com (G.S.); gvasilop@uth.gr (G.V.); 2 Cell and Gene Therapy Lab, Biomedical Research Foundation of the Academy of Athens, 11527 Athens, Greece

**Keywords:** amyloidosis, immunoglobulin light chain, diagnostic procedures, therapeutic interventions

## Abstract

Light-chain amyloidosis (AL) is a disease of protean manifestations due to a wide spectrum of organs that can be affected. The disorder is caused by the deposition of an extracellular amorphous material, the amyloid, which is produced by malignant plasma cells. The latter usually reside in the bone marrow; plasma cell infiltration is often low, in sharp contrast to what we observe in multiple myeloma. The disease may run below the physician’s radar for a while before clinical suspicion is raised and targeted tests are performed. In this short review, we try to answer most of the questions that a practicing physician may ask in a relative clinical setting. The text is formed as a series of reader-friendly questions that cover the subject of AL amyloidosis from history to current therapy.

## 1. Introduction

Amyloid refers to a substance that is similar but not identical to “amylo”, a word of Greek origin that means starch. Amyloids are proteins that have altered physicochemical properties compared to their normal counterpart, resulting in the accumulation of the material in various tissues. Each amyloid has a core substance that is a fibrillar protein from which nonbranching polymers are formed that alter the function of cells and tissues as a whole. There are a number of diseases associated with the deposition of amyloid and a number of diverse symptoms associated with the presence of amyloid resulting in major confusions that were gradually resolved when, in the late 1970’s, the chemical analysis of amyloids was clarified and a consensus nomenclature evolved. Accordingly, each disease entity that has amyloid deposition has an acronym from the letter A (for amyloid) and from the kind of amyloid present; for example, AL amyloidosis refers to amyloidosis that involves the light chains of immunoglobulins.

Amyloidosis can be either localized or systemic; the former is characterized by single organ involvement at the site of amyloid production while the latter refers to disorders where amyloid production affects distant organs generating protean disease manifestations [1]. The systemic amyloidosis category groups the following entities (with the involved protein in parentheses): AL amyloidosis (immunoglobulin light chain), ATTR amyloidosis (transthyretin protein both familial and senile), and ABeta2M amyloidosis (beta 2 microglobulin).

In this short review, specific aspects of AL amyloidosis are analyzed in the form of questions and answers (Q&A). Since AL amyloidosis is a rare disease [1] there has to be a significant degree of suspicion in the uninitiated physician in order to direct their thoughts to the diagnosis of the disease. In addition, in a systemic disease, as is the case in AL, symptoms from different organs are often mild, and connecting the dots is not always a straightforward process. To this end, we wish to provide the reader with a roadmap where all diagnostic bifurcations and ambiguities are addressed in the form of Q&As.

## 2. Historical Background

**Q1: When was the term amyloid coined?**

It seems that the renowned pathologist R. Virchow was on the right side of history, although by mistake! In a report dated 1856, he reported that the material found in degenerate CNS tissues stained positive with an iodine reaction that stains sugars; he then argued that this amorphous material was similar to starch, thus amyloid [2]. Back then, in the stone age of pathology, two other terms were also popular in describing this material, waxy and lardaceous, due to its bacon-like appearance. A report on the nature of the so-called lardaceous disease pointed out that it was of albuminous origin, arguing against the starch theory [3]. However, the sheer volume of Virchow’s authority as a prominent pathologist and the reproducible histochemical reaction with iodine were sufficient for the final prevail of the term amyloid in the scientific literature [4]. What iodine apparently stained were the various sugars that amyloid attracts on the process of polymerization.

**Q2: When was amyloid linked to plasma cell disorders?**

This, however, was not a Virchow first. It was a report in 1867 [5] that linked the presence of amyloid in heart and kidneys with multiple bone fractures where normal bone tissue was replaced by a red-greyish substance containing small nucleated cells, apparently plasma cells by today’s knowledge. At this point, it must be mentioned that in those early days of pathology, amyloid was reported most commonly in prevalent chronic infectious diseases such as tuberculosis and syphilis and much less often in neoplastic disorders.

**Q3: When was Congo Red baptized as the holy grail of amyloid detection?**

Although Congo red was discovered in 1883, it was not until 1922 that Dr Bennhold used it to stain amyloid [6]. A few years later, in 1927, Divry and Florkin, at the University of Liege, reported on the birefringence of the Congo red-stained amyloid under polarized light; in this setting, red turns into a characteristic apple green that is hard to miss [7]. Central to the specificity of the stain is the β-pleated sheet structure of amyloid. This method has become the standard technique for amyloid detection although it has to be noted that not all amyloids are created equal since a significant number of them do not stain positive for amyloid. This seems to be linked to the fact that amyloid peptides occasionally form amorphous aggregates instead of fibril polymers, in effect staining negative for Congo red [8].

**Q4: Does Congo Red come all the way from Congo?**

This is hard to say. Congo red belongs to the group of aniline dyes that were discovered accidentally in 1857 by WH Perkin in the UK and started their career as textile dyes. The problem with aniline dyes was their temporary nature since they used to wash out; in 1883, this issue was solved by the German chemist P. Böttinger, who made a brilliant stable red dye that he immediately patented [9]. No signs of Congo yet. However, in 1885, a major convention was held in Berlin that settled colonial disputes over the Congo basin, an area that was considered a source of exotic materials. The dye was then renamed Congo red as a pure marketing move to capture the attention of the wider public. A similar case was the Coomassie (or Kumasi) blue used still today to stain proteins and named to commemorate the British occupation of a part of Ghana.

**Q5: When was the immunoglobulin light chain linked with amyloidosis?**

The first reports linking the Bence Jones protein (an old term for immunoglobulin light chains in urine) to amyloidosis date back to 1931 [10] and the unambiguous association of amyloidosis with cases of myeloma must be credited to RA Kyle in a report from 1961 [11]. The exact connection between amyloid and myeloma came in 1971 when it was shown convincingly that amyloid fibrils from a couple of patients were portions of the light immunoglobulin chains [12]. Further on, the in vitro production of amyloid was also reported by the pepsin digestion of Bence Jones protein from two myeloma patients who did not have clinical amyloidosis [13]. These data argued for additional structural changes required for the in vivo generation of amyloid and the full spectrum of amyloidosis disease. Overall, our current understanding is that amyloid consists of the variable portion of the immunoglobulin light chains, it is extracellular histologically, and has a detrimental effect on the function of the involved tissue, as presented below.

## 3. Disease Manifestation

Signs and symptoms of the disease can be misleading and inconclusive. An example of such a case from our practice is the following: a 69-year-old female with an unremarkable medical history presented with weight loss (25 kg in 8 months), loss of appetite, colic pains, fatigue, anemia, and signs of heart failure. She had undergone a BM biopsy, but CR staining was not requested although her plasma cell infiltration was around 15%. A heart u/s was highly suspicious and upon request, CR staining turned out positive in the BM biopsy. AL amyloidosis is nicknamed as the great impostor due to the plethora and heterogeneity of symptoms that reflect the multiorgan involvement. It is for this very reason that it takes, on average, seven months before a diagnosis is confirmed [14]. The organs most often involved include the heart, the kidneys, the skin, the peripheral and/or autonomic nervous system, and the gastrointestinal tract. In the following lines, a detailed description of organ involvement is outlined in the form of Q&As.

**Q6: What is the most commonly affected organ in AL amyloidosis?**

The heart is affected in 75% of confirmed cases [15]. The most common presenting symptoms are fatigue, weakness, and decreased exercise tolerance [16]. During clinical examination, there are usually signs and symptoms of diastolic heart failure because of restrictive cardiomyopathy. In more advanced cases, atrial fibrillation may be present, which further contributes to clot formation and systemic embolization. Specific findings include the following:

- The electrocardiogram shows diffuse low voltage QRS (<5 mm height), QRS deviation, LBBBs, and pseudo-infarction.
- The echocardiography test reveals biventricular hypertrophy, interventricular septal hypertrophy, biatrial dilatation, decreased longitudinal strain in the mid and basal wall with relative sparing of apical function.
- NTproBNP is the most sensitive marker (100% sensitivity) for cardiac involvement in AL amyloidosis and constitutes an important factor in survival and prognosis [17].
- Cardiovascular magnetic resonance (CMR) imaging with late gadolinium enhancement (LGE) shows circumferential subendocardial LGE in AL amyloidosis. It is used for the diagnosis of cardiac AL amyloidosis but also as a prognostic tool [18].
- Endomyocardial biopsy detects and recognizes of the type of amyloid with high accuracy. When there is suspicion of cardiac amyloidosis, the endomyocardium should be biopsied rather than peripheral organs [19].

Complementary tests:

- Myocardial PET/CT shows enhancement of the radiotracer (technetium) due to the amyloid binding; currently, it is only used as a research tool [16].
- Cardiac MRI is used mainly for prognostic and monitoring purposes. It shows a diffuse decrease in the T1 and T2 signal intensity of myocardium. Moreover, myocardial native T1 and T2 are elevated. T2 elevation is associated with worse prognosis.

Overall, AL cardiac amyloidosis is a condition with poor prognosis, with an estimated median overall survival of 6–12 months, if left without treatment [20].

**Q7: How does AL amyloidosis affect the kidneys?**

In AL amyloidosis, a plasma cell clone produces a misfolded, monoclonal light immunoglobulin chain that often has the propensity to be deposited in the glomerulus, a feature that is more common with lambda chains due to their potential to dimerize [21]. Overall, light chain deposits constitute around 80% of all renal cases of amyloid deposition. Kidneys are affected in about 50% of patients, as evidenced by lower-than-normal GFR and proteinuria; the latter, when excessive, can lead to nephrotic syndrome and anasarca that further deteriorates the performance status of the patient. The severity of kidney dysfunction varies, but the percentage of patients who eventually develop end-stage renal failure is high [22].

**Q8: Could a gastroenterologist be the first doctor to suspect AL amyloidosis?**

The liver and the gastrointestinal tract can be affected in 15% and 5% of the confirmed cases, respectively. Patients with GI involvement usually have more advanced disease and may have other organs involved simultaneously [23]. Patients might present with GI bleeding, signs and symptoms of malabsorption (weight loss, diarrhea, and steatorrhea) [24], and protein-losing gastroenteropathy with excessive hypoalbuminemia [25]; more mild cases present with symptoms of chronic dysmotility such as constipation and/or diarrhea, nausea, vomiting, abdominal pain, and bloating [26]. The endoscopic findings in such cases are not specific, but if suspected, blind biopsies should be taken from various sites. In addition, a careful histopathologic evaluation should be sought where the suspicion for amyloid deposition must be clearly stated in the clinical information sheet that accompanies the specimens. If this suspicion is not raised, amyloid deposition can be easily missed even by experienced pathologists.

Beyond the intestine, liver involvement is not so uncommon and has an incidence that varies from 7% during disease course to 70% at autopsy reports. When the liver is involved, it usually manifests with hepatomegaly, jaundice, and, rarely, cholestasis with increased alkaline phosphatase. There have been occasional reports of severe liver failure [27], but overall, liver involvement usually runs a subclinical course.

**Q9: What other symptoms can a patient with AL amyloidosis demonstrate?**

As discussed above, fibril deposition can occur in every organ system, hence the heterogeneity of the presenting symptoms. The patient might suffer from dyspnea and hoarseness if the disease affects the lungs and the airways, including the vocal cords. In such cases, the imaging tests might reveal pleural effusions, pulmonary nodules and cysts, and finally, diffuse alveolar deposits. If the peripheral and/or the autonomic nervous system are involved, the patient develops peripheral neuropathy with paresthesia, while orthostatic hypotension, erectile dysfunction, and sweating abnormalities are more prominent when the autonomous path is affected [28]. Manifestations of soft tissue disease include carpal tunnel syndrome, trigger finger, and bicipital tendon rupture. Other symptoms may include macroglossia, periorbital purpura, and bleeding diathesis [28]. It has to be mentioned that from our practice, the latter physical findings, although considered disease hallmarks in various textbooks, are less often observed due to the earlier detection of the disease; practically, their absence should not discourage diagnostic effort.

## 4. Diagnostic Procedures and Differential Diagnosis

The worse risk factor in the overall survival for the AL amyloidosis patients is the delayed diagnosis of the disease. The reason behind this dismal observation is often the subtle clinical and biochemical findings that do not raise suspicion and timely interventions. In effect, the delayed diagnosis results in irreversible organ damages that are often not amenable to treatment. The symptoms of disease onset depend on the organs involved and can be nonspecific; beyond the common finding of heart failure, one may encounter organomegaly, GI symptoms due to a dysfunctional autonomous neural system, weight loss due to malabsorption, unexplained fatigue, and incidental skin bruising. The tests that allow for diagnosis of amyloidosis are relatively accessible, and clinical doctors should ask for them when suspicion is raised. High-risk patient populations such as MGUS patients should be closely monitored for relative markers such as NT-proBNP and albuminuria [29]. Moreover, cases of AL amyloidosis misdiagnosed as scleroderma [30] or jejunal mass [31] have been reported because of the nonspecific symptoms. In similar cases, diagnosis and therapy have been significantly delayed.

**Q10: What is the diagnostic algorithm?**

When AL amyloidosis is suspected, the initial work up includes noninvasive and invasive procedures.

Noninvasive procedures: These include a complete blood count with serum biochemistries. Specific tests must include serum and 24 h urine protein electrophoresis with immunofixation, beta2-microglobulin, and serum-free light-chain quantitation. Cardiac evaluation should include Troponin-T, NTproBNP, ECG, and echocardiography. Finally, if signs of neural involvement are present, electromyography and nerve conduction studies should be part of the initial workup (Table 1). 

Invasive procedures: These tests should be applied as reflex tests, i.e., performed in a sequential fashion (Table 1). Bone marrow (BM) biopsy is mandatory for the evaluation of PC infiltration and the detection of the amyloid. If amyloid is not detected in the BM, then comes abdominal fat aspirate, and if this is also negative, amyloid deposits should be sought in any organ where clinical suspicion for involvement has been raised [28,32]. These could be the kidneys, the lung, an enlarged lymph node, or the GI tract.

The cornerstone of diagnosis is the detection of amyloid deposition on the examined tissues using thioflavin dyes and Congo red. However, not all amyloids stain with Congo red, and often, the histology reports describe the deposition of “amorphous eosinophilic material”; in these cases, if clinical suspicion is high, the diagnosis can be made with certainty. It should be mentioned that, a small number of false positives, Congo red stain has been reported due to the fact that the stain binds to other native proteins such as cytoskeletal proteins [33]. Where possible, diagnosis can be unquestionably established with mass spectrometry, which is the gold standard for disclosing the kind of amyloid chain involved [34].

**Q11: Is AL amyloidosis the only form of amyloidosis?**

Light-chain amyloidosis should be distinguished from other forms of amyloidosis and other types of monoclonal immunoglobulin deposition diseases [35]. The differential diagnosis includes:

- Wild-type ATTR amyloidosis (ATTRwt), an age-related amyloidosis, caused by wild-type transthyretin deposition in the myocardium without immunoglobulin light chains [36].
- Hereditary amyloidosis can be divided into:
  - Hereditary amyloid transthyretin amyloidosis (ATTRv) caused by systemic deposition of mutated form of transthyretin (TTR) amyloid fibrils inherited as an autosomal dominant trait. The main ATTRv phenotypes include ATTR cardiac amyloidosis, ATTR leptomeningeal/CNS amyloidosis, and ATTR amyloidosis polyneuropathy [37].
  - Non-ATTR hereditary amyloidosis consists of other inherited gene mutations (apolipoprotein AI and AII, fibrinogen Aa, lysozyme, gelsolin, and cystatin C)
- AA amyloidosis: this is related to a chronic inflammatory condition and is characterized by the extracellular deposition of fibrils derived from the serum amyloid A (SAA) protein [38].

**Q12: What is the role of Tc-99m scintigraphy in the diagnosis of amyloidosis?**

Tc-99 m scintigraphy is highly accurate for the diagnosis of ATTR, with a positive predictive value of 100% when a monoclonal light chain is not detected. In contrast, its diagnostic value for AL amyloidosis is low, with both sensitivity and specificity being below 50%. It is performed with Tc-99m DPD, Tc-99m HMDP, and Tc-99m PYP. The radiotracers bind to microcalcifications in the amyloid fibrils. The density of microcalcifications is greater in ATTR amyloidosis than in AL amyloidosis and so this method has high detectability for ATTR. This imaging technique can be false negative for ATTR when a specific TTR mutation that does not allow myocardial uptake is present [39].

**Q13: What is the diagnostic approach for distinguishing ATTR and AL amyloidosis?**

ATTR amyloidosis is probably the leading differential diagnosis for AL amyloidosis. AL amyloidosis and ATTR both are treatable, but the therapy is vastly different, so it is crucial they be distinguished. Patients with clinical symptoms suspicious for cardiac amyloidosis should undergo assessment for the presence of monoclonal protein in serum and urine by serum PEP with immunofixation, urine PEP with immunofixation, and serum κ/λ free light chains ratio. If monoclonal protein is detected, a hematologist should continue the evaluation. The absence of monoclonal protein has a negative predictive value of 99% for AL amyloidosis and, in conjunction with a positive cardiac scintigraphy (Grade 2–3 myocardial uptake), can establish the diagnosis of ATTR [40]. When diagnostics dilemmas exist, endomyocardial biopsy should be performed in specialized centers. If ATTR amyloidosis is confirmed, further genetic testing is needed to confirm or exclude a variant in the TTR gene and so to diagnose ATTRwt from ATTRv [41]. In the case of ATTRv, there is also positive family history.

**Q14: How is AL amyloidosis related to other plasma cell disorders?**

AL amyloidosis and monoclonal plasma cell disorders such as multiple myeloma (MM) and monoclonal gammopathy of undetermined significance (MGUS) are both characterized by the presence of malignant plasma cells in the bone marrow. Excess plasma cell infiltration in the bone marrow, bone fractures with hypercalcemia, and M-protein deposition in the kidneys are the leading causes of morbidity in multiple myeloma. In amyloidosis, plasma cells do not accumulate in high numbers; the typical manifestations of MM are missing but the PCs secrete an abnormal monoclonal immunoglobulin protein that can form amyloid, which leads to deposits. AL amyloidosis usually develops in a small percentage of MM/MGUS patients, usually less than 10%. Thus, when signs of heart failure or of other disease manifestations not typical of MM develop in MM/MGUS patients, the possibility of AL amyloidosis should be investigated [42].

**Q15: Are there cases of systemic monoclonal immunoglobulin deposition disease (MIDD) that are not AL amyloidosis?**

Beyond the classical light-chain amyloidosis, there are another two MIDDs that are exceedingly rare: light-chain deposition disease (LCDD) and heavy-chain deposition disease (HCDD). For both of them, the culprit is a clonal plasma cell expansion. LCDD is the deposition of monoclonal light chains in multiple organs. It is characterized by the presence of a nonamyloid immunoglobulin light chain, which, unlike amyloid, does not stain with Congo red; they are more often kappa than lambda and do not arrange as fibrils but as granular structures in the tissues involved. The clinical course of the disease is similar to AL amyloidosis [43]. HCDD is also a rare B cell proliferative disorder that has clinical characteristics similar to AL amyloidosis and LCDD. The deposits in HCDD are intact or truncated heavy chains that also do not stain positive with Congo red [44]. 

## 5. Therapeutic Interventions

A first and very important step in designing the therapeutic approach is the assessment of a patient’s frailty status, since the young and fit can receive more intense therapies such as high-dose melphalan followed by hematopoietic cell transplantation (HCT). There is no specific frailty score for amyloidosis, although age and preexisting comorbidities can provide a rough guide in the decision-making process. Beyond frailty, the characteristics of the malignant plasma cell clone and the kind of the organs involved are equally significant factors for the selection of treatment.

**Q16: How are the patients risk-stratified?**

The disease stratification most commonly used is based on the Standard Mayo Clinic staging system [45,46]. From 2012, Mayo Clinic has set the NT-proBNP, cardiac troponin T(cTnT), cardiac TroponinI (cTnI), and high-sensitivity troponin (hs cTnT) markers in the staging system, as outlined in Table 2.

The cardiac involvement and the dFLC burden is the most significant factor for prognostication in AL amyloidosis. Patients with stage I AL amyloidosis have an overall survival of 7.8 years, stage II have 3.4 years, stage III have 14 months, and stage IV have 5.8 months [45].

Furthermore, the patients are risk-stratified in three categories: **(i)** low-risk eligible for HCT, **(ii)** intermediate-risk, and **(iii)** high-risk, both ineligible for HCT. A mere 20% of the patients meet the eligibility criteria outlined in Table 3. In practical terms, eligibility requires sufficient cardiac, renal, and liver function. If certain measurable criteria are not met, HCT has an unacceptable treatment mortality rate and should be avoided [48]. The intermediate- and high-risk patients (80% patients in total) are ineligible for HCT and should be treated with less intense regimens.

**Q17: What is the aim of the therapy?**

The aim of the treatment is to obliterate the production of the amyloidogenic light chains by the plasma cell (PC) clone in order to rescue organ function from further amyloid deposition and provide the patient time to recover. Such PC-directed therapies were originally developed for the management of multiple myeloma and have been adjusted for the treatment of AL amyloidosis. Early and significant reduction in amyloidogenic light chains is highly desirable, because it is associated with prolonged progression-free and overall survival [49]. However, it should be mentioned that in many cases of AL amyloidosis, the neoplastic PC infiltration in the BM is low, and intense treatments such as those administered in MM may be unnecessary and even harmful.

**Q18: How is response defined?**

The assessment of the response to treatment has two components: the hematological and organ response. Since AL is caused by light-chain immunoglobulins, the difference between involved and uninvolved free light chains (dFLCs) has evolved as the method of choice to evaluate clonal disease response, since it provides more accurate assessment compared to the ratio of the free light chains. Four (4) hematological response categories have been defined [50]:

- Complete response (CR), when serum and urine immunofixation is negative with normalized ratio of FLC;
- Very good partial response (VGPR), when the difference of dFLC is <40 mg/L;
- Partial response (PR), when dFLC are decreased by 50%;
- No response, when none of the above is achieved.

As far as the heart is concerned, response is defined as a decrease in NT-proBNP by 30% and by 300 ng/L from baseline at the end of therapy. A note of caution: when evaluating cardiac biomarkers, other factors that affect them, such as renal failure or treatment with immunomodulatory agents, should be taken into account. 

Renal response is defined as a reduction of >30% from baseline proteinuria in the absence of a more than 25% rise in creatinine over baseline. Finally, hepatic response is defined as a reduction of more than 30% from baseline serum alkaline phosphatase levels [51].

**Q19: What is the current standard of care?**

Once the diagnosis of AL has been firmly established, treatment should be started promptly. A recent and major improvement in the treatment of AL has been the introduction of the anti-CD38 antibody daratumumab. Based on the results of the Phase III Andromeda study [52], deeper responses were achieved in newly diagnosed patients who received daratumumab plus bortezomib, cyclophosphamide, and dexamethasone (DaraCyBorD) compared to those who received plain CyBorD. The hematologic complete response rate was 53% for the D-CyBorD arm vs 18% for the CyBorD arm. Organ responses were also significantly higher in the DaraCyBorD arm; cardiac and renal responses were 41% vs. 22% and 53% vs. 24%, respectively. This study is considered as a paradigm shift in the treatment of AL amyloidosis [53]. As a result, daratumumab and proteasome inhibitors remain the backbone of any frontline therapy. A similar paradigm shift in the treatment of AL was the addition of proteasome inhibitors such as bortezomib; in the EMN-03 trial that compared the addition of bortezomib to melphalan and dexamethasone, a significant improvement in overall response was recorded (81% vs. 57%) [54]. Summarizing, for stage I patients, eligible for ASCT, DaraCyBorD is recommended as induction therapy. The same regimen is also indicated for all subsequent stages (II and III) with modifications (such as dose reductions) based on comorbidities. In addition, Velcade–melphalan–dexamethasone, dose-modified CyBorD, or daratumumab monotherapy are all possible therapeutic options [55]. Finally, immunomodulatory (IMiDs) agents such as thalidomide and its derivatives have had their share in AL trials but have never emerged as significant players in the battle against this devastating disorder. The renal toxicities associated with their use have limited their wide application in a disease that involves the kidneys in nearly 50% of cases. Newer PIs (ixazomib and carfilzomib) have shown activity in small PhI/II trials, but long-term data are pending.

**Q20. What is supportive therapy in AL amyloidosis?**

Supportive therapy has a fundamental role in the management of AL amyloidosis. The aim is to improve quality of life, relieve symptoms, and sustain organ function. The main target of supportive treatment is diuretic therapy and salt restriction in order to reduce fluid retention. Angiotensin-converting enzyme (ACE) inhibitors and angiotensin receptor blockers are frequently used to minimize proteinuria. However, ACE inhibitors are generally poorly tolerated because of hypotension. Gabapentin or pregabalin have a place in management of neuropathic pain. Furthermore, nutritional support and adequate caloric intake are important in order for albumin to be maintained at a satisfactory level [56]. 

## 6. Novel Therapies

**Q21. Are there antibodies that target amyloid deposits?**

Although many patients achieve hematological response, the organ responses either lag behind or are entirely negligible, owing to the inability of the endogenous “waste management”. In a complementary manner to anti-PC treatment, agents that target and eliminate amyloid deposits are a promising therapeutic avenue. Three such anti-AL antibodies have been developed to date.

- **Birtamimab (NEOD001)** is humanized monoclonal antibody that clears the deposited amyloid fibrils by phagocytosis. It interacts with cryptic epitopes that are exposed on misfolded molecules and clears the amyloid from the organs and the bloodstream. A post-hoc analysis from the phase III VITAL showed increased OS for patients with advanced AL amyloidosis. An ongoing double-blind, phase III study, AFFIRM-AL, was designed to confirm the finding of VITAL [57].
- **Anselamimab (11-1F4)** is also a monoclonal antibody that binds to a conformational epitope in the N-terminal of both κ- and λ-amyloid deposits in the organs. Phase 1a/1b study data showed that anselamimab is well tolerated and could improve organ function, especially of the heart [58]. Moreover, there two ongoing randomized phase 3 studies of anselamimab combined with standard-of-care therapy in advanced AL amyloidosis (NCT04512235, NCT04504825) that could alter the treatment landscape [59].

**Q22. Could antibody–drug conjugates be used in treatment amyloidosis?**

Antibody–drug conjugates (ADC) are currently used in the treatment of R/R MM. One such product is belantamab mafodotin, a humanized IgG1 monoclonal antibody targeting BCMA+ cells, where it delivers its cargo, auristatin-F, a microtubule-disrupting agent called mafodotin. This is the first-in-class ADC to be used in plasma cell disorders and its activity on AL patients has been explored in an open-label, multinational, phase 2, EMN27 study [60]. The early data show a 53% hematological response but lower responses in affected organs; however, one has to consider the short treatment duration and the high number (>3) of prior treatments these patients had received. Importantly, no novel AEs have emerged.

**Q23. Will CAR-T be the answer for Amyloidosis?**

In the last two years, the FDA has approved two CAR-T products targeting BCMA for patients with refractory MM after three lines of treatment [50,61]. Reports on the use of these products in AL patients have been scarce in the literature. It is worth mentioning a study from Israel where the investigators used a homemade anti-BCMA CAR-T to treat eight AL patients with an average of five different lines of therapy [62]. According to the study, 5/8 achieved a complete hematologic response, 2 achieved a very good partial hematologic response, and 1 achieved a partial hematologic response. It has to be noted that organ responses were observed in 6/8 patients, a fact that supports the further exploration of this treatment modality in the AL field.

## 7. Discussion

Light-chain amyloidosis is not a death sentence anymore. Disease awareness has been raised and diagnosis is currently made in earlier stages with a clear effect in overall survival and quality of life. This trend should be further supported by targeted education in primary health care physicians and nurses. Suspicion for AL should be raised in cases where subtle symptoms from different organs need a “connecting glue”; a prepared mind can set the pieces together and come up with the diagnosis, which remains elusive if AL is not part of the differential. The available proteasome inhibitors and anti-CD38 monoclonal antibodies have changed the course of the disease. Further improvements are awaited with the novel bispecific T-cell engagers, while the holy grail of AL treatment, i.e., the inhibition of amyloid formation, has yet to be discovered. 

## Figures and Tables

**Table 1:** Diagnostic procedures in the initial workup for AL amyloidosis.

Noninvasive	Invasive
CBC	BM biopsy
Serum biochemistries	Fat pad biopsy
β2 microglobulin	Colonoscopy *
Serum PEP	
Urine PEP	
Urine 24 h protein	
FLC (serum and urine)	
TnT and NTproBNP	
ECG	
EMG * Tc-99m scintigraphy	

CBC: complete blood count; PEP: protein electrophoresis; FLC: free light chains; ECG: electrocardiogram; EMG: electromyogram; * when suspicion is raised for organ involvement.

**Table 2:** Mayo Clinic staging system [47].

Variables and Cutoffs	Stages
NT-proBNP, 1800 ng/L	Stage I: all markers below the cutoffs
dFLC, 180 mg/L	Stage II: one marker below the cutoffs
cTnT, 0.025 ng/mL	Stage III: two markers below the cutoffs
(cTnl, 0.1 ng/mL or hs cTnT, 40 ng/L)	Stage IV: all markers above the cutoffs

**Table 3:** Eligibility criteria for HCT in patients with systemic light-chain amyloidosis [46].

Kind	Value
Age	<70 years
ECOG PS	0–2
NT-proBNP	<5.000 ng/L
Troponin	<0.06 ng/mL
eGFR	>50 mL/min per 1.73 m^2^
NYHA class	<III
Ejection fraction	>45%
Systolic BP	>100 mmHg
Bilirubin	<2 mg/dL
DLCO	>50%

PS: performance status; eGFR: estimated glomerular filtration rate; DLCO: diffusion capacity of carbon monoxide.

## Data Availability

Not applicable.
